# Participatory and Conventional Investigation of Tick Infestation in Camels and Cattle of Somali Pastoral Areas, Eastern Ethiopia

**DOI:** 10.1155/2023/5840827

**Published:** 2023-12-18

**Authors:** Mohamoud Mohamed Jama, Hassan Abdi Hussein, Shaban Mohamed Abdi, Teka Feyera

**Affiliations:** College of Veterinary Medicine, Jigjiga University, P.O. Box 1020, Jigjiga, Ethiopia

## Abstract

Ticks are a common parasite that affect many animals by causing slowed growth, reduced milk output, and financial losses for industries that depend on animal hides and skins. From June to December 2017, participatory and conventional investigations on tick infestation in camels and cattle were conducted in Kebribayah and Afdem districts of Ethiopia's Somali Regional State. The aim of this study was to determine the prevalence and density of ticks in these animals and establish strategic control measures to enhance livestock productivity and livelihoods in pastoral areas. The current study found that the prevalence of tick infestation in Kebribayah and Afdem districts was 83.3% and 86.8%, respectively. *Rhipicephalus pulchellus* (48.9%) was identified as the most common tick species in camels and cattle, followed by *Amblyomma gemma* (26.3%), *Hyalomma truncatum* (11.6%), *Amblyomma lepidum* (6.7%), and *Amblyomma variegatum* (6.5%). Among the variables considered, age and body condition score were significant risk factors (*p* < 0.001). Tick density varied depending on the recorded months and seasons (*p* < 0.001), with the highest mean tick density occurring in November (32.69 ± 21.750) and during the wet season (28.56 ± 19.750). Livestock owners in Kebribayah and Afdem ranked topical acaricide application as the most effective tick control method, followed by ivermectin injections, with the traditional hand removal method being the least effective. These rankings were consistent across both districts, and there was moderate agreement among livestock keepers from both regions regarding the best method. Afdem livestock keepers had slightly weak agreement on high tick burden in spring (*W* = 0.475, *p* = 0.127), and Kebribayah livestock keepers showed slightly strong agreement in tick burden across seasons (*W* = 0.700, *p* = 0.038), with spring having a significantly higher burden than winter. Consequently, participatory appraisal indicated that ticks were important and prevalent ectoparasites in the study area. Finally, strategic tick control appropriate for specific management and production environments should be implemented biannually in wet seasons.

## 1. Introduction

The increasing adoption of camels by pastoralists in Ethiopia is driven by their unique adaptability and resilience, offering a promising livelihood diversification strategy to enhance food security amidst the challenging environmental conditions [1]. Similarly, cattle are kept for various purposes, with milk being the highest priority, followed by meat and breeding for future sales, as a means of saving income for pastoralists [2].

Ticks (Ixodoidea) are the most prevalent ectoparasites that belong to the Acari order, which also includes mites and spiders. Ticks are parasitic arachnids that feed on the blood of mammals, birds, and reptiles. They can transmit a variety of diseases to animals, including babesiosis, anaplasmosis, heartwater, and Lyme disease. Ticks can also cause blood loss, skin irritation, and reduced hide quality. Ticks exhibit dioecious reproduction, meaning that they have separate sexes: male and female. They are classified as one-, two-, or three-host ticks based on the number of hosts required completing their life cycle [3]. Ticks can have a detrimental impact on livestock health and productivity. In some cases, tick infestations can lead to slow growth rates, decreased milk output, and even mortality. Moreover, skin bites from ticks can reduce the quality of hides and skin, causing significant losses in revenue from exports [4].

Hard ticks are vectors of harmful pathogens of rickettsia, bacterial, viral, and protozoan origin, which cause serious infectious diseases in humans and livestock [5]. Tick-borne diseases (TBDs) are the most significant constraints on livestock production systems in Ethiopia [6]. In Ethiopia, ruminant ectoparasites are responsible for significant financial losses for small-scale farmers, the tanning sector, and the nation as a whole because of animal mortality, decreased output, degradation, and the rejection of hides. Additionally, economic losses are incurred in control costs through chemotherapy, vaccination, and tick control using acaricides. Ticks are considered the most economically significant ectoparasites for livestock in the tropics, particularly in sub-Saharan Africa [7]. Ticks that are considered crucial to the health of domestic animals in Africa comprise approximately seven genera and forty species. The most common tick genera in Ethiopia are *Amblyomma* and *Boophilus*, followed by *Haemaphysalis*, *Hyalomma*, and *Rhipicephalus* [8].

Pastoral communities in Africa live in some of the least developed and harshest environments in the world. Livestock herding contributes significantly to the social and economic well-being of these communities [9]. Communities in the Somali pastoral area rely heavily on camels and cattle for their livelihoods, and the area is characterized by the extensive grazing of livestock in communal ranges. Owing to repetitive cycles of drought, transhumance has been adopted as a coping strategy for survival. In the rainy season, livestock is kept in enclosures located closer to the permanent settlement area; however, in search of pastures and water during the dry season, pastoralists move their livestock to nearby areas and countries [10].

Ethiopia has the largest livestock population in Africa, with 56.71 million cattle, 29.33 million sheep, 29.11 million goats, 2.43 million donkeys, 0.4 million mules, 1.16 million camels, 56.87 million poultry, and 5.88 million beehives [11]. Livestock farming is an important resource for smallholder farmers in Ethiopia, providing milk, meat, skin, manure, and traction [12]. Livestock products, including live animals, meat, and leather goods, are a significant source of foreign exchange [13]. Despite the importance of livestock in the region, there is limited information from systematic studies on tick infestation in pastoral areas in the Somali Region. Additionally, government veterinary services are poorly funded, and there is a substantial shortage of veterinarians. Thus, the use of conventional disease investigation and surveillance in pastoral areas is limited because pastoralists live in transboundary ecosystems and must cross national borders to access grazing areas [14]. Consequently, local knowledge of livestock diseases has not been fully explored [15]. Hence, epidemiologists have recently modified participatory epidemiology (PE) techniques to better understand the effects of livestock diseases on pastoralists' livelihoods [16, 17]. This study is aimed at determining the prevalence and density of ticks in camels and cattle raised in the pastoral areas of the Somali Region, Ethiopia, and establish strategic control measures to improve livestock productivity and livelihoods.

## 2. Materials and Methods

### 2.1. Description of the Study Area

**Figure 1:**
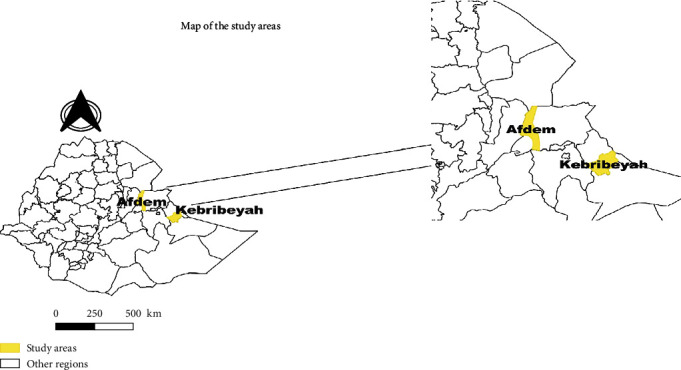
Map of the study districts.

The study was conducted in two districts (Figure 1) that represent pastoral and agropastoral livelihood zones of the region. The districts were selected based on the prevailing production system and the existence of a large livestock population. Kebribayah is 680 km east of Addis Ababa, at a latitude of 9°6′ N and longitude of 43°10′ E, with an elevation of 1686 m above sea level. The district is characterized by an arid and semiarid climate, with mean maximum and minimum annual temperatures of 29°C and 14°C, respectively, and low relative humidity, with bimodal annual rainfall ranging from 700 mm to 900 mm. The Afdem District is located 250 km east of Addis Ababa at a latitude of 10°15′ N and longitude of 41°10′ E, with an elevation of 2000 m above sea level. The district has a semiarid environment influenced by highlands that are close to and receive approximately 600 mm of rainfall per year. The average annual high temperature is 27.7°C, with a low temperature of 13.5°C. The rainy season occurs between March and June, with a short secondary rainy season from October to December. In this region, two production and grazing methods are used: pastoral herds, which travel across broad swaths of land in search of pasture and water, and agropastoral herds, which are maintained by village inhabitants and have less mobility until they are affected by drought or other situations.

### 2.2. Participatory Epidemiology

Participatory epidemiological methods were used to generate relevant data and to gather local knowledge. Accordingly, multiple tools, including informal interviews, visualization, seasonal calendars, ranking, and scoring methods, were employed.

#### 2.2.1. Key Informant Discussion

Participatory meetings and interviews were conducted with key informants to obtain a general overview of the tick infestation situation and constraints on tick control options in their villages. The key informants were selected through a process that involved recommendations from community elders and religious and tribal leaders, as well as suggestions from animal health experts and government officials. Specifically, these individuals recommended people who had extensive experience in animal raising and prolonged husbandry practice from the local area. Information from key informants was used to select participants and peasant associations (PAs) [18].

#### 2.2.2. Focus Group Discussion

Focus group discussions (FGDs) were conducted with 10–12 key respondents to understand local livestock owners' perceptions of tick infestation, seasonal tick population dynamics, control options, and trends of tick spread among animals. Twenty-four focus groups were formed from eight PAs in the districts, selected purposively based on information from a key informant discussion about prevalent tick infestation. The case definition was described to the key informants without telling them of the disease with which it was associated with a void bias. Participatory epidemiology (PE) tools, such as semistructured interviews, proportional piling, and seasonal calendars, were used to collect data during FGDs [19–21].

#### 2.2.3. Semistructured Interviews

Semistructured interviews were conducted to gain a better understanding of the perceptions of the community and beliefs regarding seasonal tick population dynamics, species composition, and tick control options and constraints in cattle and camels in the study areas. Respondents were asked to name and describe common ectoparasites affecting camels and cattle in their area, as well as the circumstances under which these ectoparasites infestations occurred, using the local language of the area (Somali). They were also probed for the ectoparasites of interest (tick infestation) with regard to its burden and risk factors and to compare the appropriate control options for tick control and the season of the year by which ticks can be controlled easily in the area.

#### 2.2.4. Proportional Piling

This tool was used to estimate the incidence of common ectoparasites in cattle and camels in Kebribayah and Afdem districts. A circle was drawn on the flip chart representing each disease, and the participants allocated 100 counters (beans, maize seeds, or stones) to each circle according to their relative importance and occurrence [16].

#### 2.2.5. Seasonal Calendar

A seasonal calendar was used to describe the seasonal variation in the prevalence and importance of the key ectoparasites. It was divided into four periods: autumn, winter, spring, and summer. The informants were given 100 objects and were asked to show the relative occurrence of tick infestation in camels and cattle during each season. When placing the objects for tick infestation against the season was complete, the group was requested to check the scores and rearrange them until they were contented with the results. More items were added one by one to the seasonal calendar and scored by the informant groups. A completed seasonal calendar comprised four seasons along the *y*-axis and a response in tick burden with respect to the seasons of the year along the *x*-axis.

### 2.3. Study Animals

The study animals were indigenous cattle (*Bos indicus*) also known as Somali zebu and camels (*Camelus dromedarius*) that were managed under an extensive production system in various agroclimatic conditions. Animals of both sexes and various age and body condition groups were randomly selected for this study. Age was determined on the basis of the owner's information and dentition. Body condition scoring (BCS) was graded as poor, medium, or good using the modified guidelines described by [22, 23].

### 2.4. Study Design

A combination of participatory epidemiology and conventional investigations of tick infestation in camels and cattle was conducted from June 2017 to December 2017 in the Kebribayah and Afdem districts of the Fafan and Sitti zones, respectively, in the Somali Regional State of Ethiopia.

### 2.5. Sample Size Determination and Sampling Method

The sample size was calculated using the formula described by Thrusfield et al. [24]. Considering the 50% expected prevalence, 5% absolute precision, and 95% confidence interval, 384 animals were required for the sampling. However, considering the vastness of the study area, 450 animals were sampled from each district, making a total of 900 camels and cattle. A multistage random sampling approach was used to collect ticks from cattle and camels in pastoral areas of the selected districts.

### 2.6. Tick Collection and Identification

A thorough search was conducted on the anatomical sites commonly preferred by ticks, including the scrotum/udder, groin, dewlap, belly, tail, leg/hoof, and neck. All attached ticks on one side of the animal's body were meticulously collected using gentle force, ensuring their intact removal to prevent decapitation. To obtain a comprehensive count for the entire body, the collected ticks were doubled. The collected adult ticks were preserved in a properly labeled plastic container containing 70% ethanol and identified using a stereomicroscope [25].

### 2.7. Data Analysis

The collected data were analyzed using the statistical software STATA version 11. The prevalence of tick infestation was assessed using descriptive statistics, and the association of potential risk factors was assessed using the chi-square (*χ*^2^) test. Agreement between different informant groups was assessed using Kendall's coefficient of concordance (*W*).

## 3. Results

### 3.1. Participatory Epidemiology

Focus group discussions were carried out in the Kebribayah and Afdem districts to explore local knowledge and information about tick population dynamics, species composition, and tick control options in camels and cattle. The study conducted four FGDs in each district, with each carried out in a different kebele. Participatory epidemiology tools, such as semistructured interviews, proportional piling, and seasonal calendars, were used during the FGDs. Eight focus groups were conducted, with 10–12 key respondents participating in each (Table 1) [20, 21].

The results of the proportional piling exercise indicated that ticks (Shilin) were the most significant ectoparasites of camels and cattle in both Kebribayah and Afdem districts, with mean scores of 57.0 ± 6.218 and 63.50 ± 5.508, respectively (Table 2). Mites (Cadho) ranked second with mean scores of 28.50 ± 1.915 and 24.0 ± 5.657 in both districts, followed by lice (Injir) at 14.50 ± 5.260 and 15.0 ± 10.00, respectively. The overall ranking of the ectoparasites was consistent between the two districts, with strong agreement (*W* = 1, *p* < 0.018 for Kebribayah and *W* = 0.813, *p* < 0.039 for Afdem).

Proportional piling exercises were used to measure the tick burden of various age groups and sexes in camels and cattle and to rank them accordingly, as indicated in Table 3. For camels in Kebribayah, young camels (mean ± SD = 23.00 ± 6.27, rank #2) had a higher tick burden than she camels (mean ± SD = 16.25 ± 4.79, rank #3) and adult camels (mean ± SD = 9.50 ± 4.20, rank #6). Similarly, for cattle in Kebribayah, young cattle (mean ± SD = 23.50 ± 4.73, rank #1) had the highest tick burden, followed by cows (mean ± SD = 16.50 ± 4.73, rank #4) and adult cattle (mean ± SD = 11.25 ± 2.99, rank #5). The results indicated a significant agreement between the two districts for both Kebribayah (*W* = 0.788, *p* = 0.008) and Afdem (*W* = 0.640, *p* = 0.025).

Tick control options include the application of topical acaricides (spraying, pour-ons, and dipping), ivermectin injections, manual removal, and integrated control of ticks. According to the rankings, topical acaricide usage as a tick control method was perceived to be the most effective by both Kebribayah (mean ± SD = 42.50 ± 6.46, rank #1) and Afdem (mean ± SD = 40.00 ± 4.082, rank #1) livestock keepers. The use of the ivermectin tick control method was ranked as the least effective by Kebribayah (mean ± SD = 20.00 ± 9.13, rank #3) and the second least effective by Afdem (mean ± SD = 23.75 ± 4.787, rank #2) livestock keepers. Manual removal and integrated control of ticks were ranked in the middle, with some variation in perceived effectiveness across the districts (Table 4). Statistical analysis indicated a moderate level of agreement between Kebribayah and Afdem livestock keepers regarding the perceived effectiveness of tick control options (*W* = 0.678, *p* = 0.043 for Kebribayah and *W* = 0.711, *p* = 0.036 for Afdem).

In Kebribayah, the highest mean score and ranking were for spring with a score of mean ± SD = 51.50 ± 8.888, while winter had the lowest mean score and ranking (mean ± SD = 11.25 ± 6.292). A similar trend was observed in Afdem, with spring having the highest mean score and ranking (mean ± SD = 45.00 ± 10.801) and winter having the lowest mean score and ranking (mean ± SD = 13.00 ± 7.257). Statistical analysis revealed no significant difference in tick burden across seasons for Afdem (*W* = 0.475, *p* = 0.127). However, for Kebribayah, there was a significant difference in tick burden across seasons (*W* = 0.700, *p* = 0.038), with spring having a significantly higher tick burden than winter (Table 5).

### 3.2. Prevalence of Tick Infestation

The distribution and abundance of different tick species found in camels and cattle during the first round of tick collection from late June to November 2017 revealed that five species of ticks belonging to the three genera were collected in the study area. *Rhipicephalus pulchellus* was the most prevalent, with a prevalence of 48.9%. This was followed by *Amblyomma gemma*, which had a prevalence of 26.3%. *Hyalomma truncatum* had a prevalence of 11.6%, whereas *Amblyomma variegatum* and *Amblyomma lepidum* were less prevalent, with prevalence rates of 6.5% and 6.7%, respectively (Table 6).

According to this study, tick infestation is prevalent in camels and cattle in the study area, with an overall prevalence rate of 85.1% among the 900 animals examined. As shown in Table 7, the prevalence of tick infestation in relation to the different risk factors was calculated. Among the variables considered, age and BCS were significant risk factors (*p* < 0.001), with adult animals and those with poor BCS having a higher prevalence of tick infestation, with 90.8% and 91.6% tick infestation, respectively. There was no significant variance in tick infestation prevalence across animal origin, sex, and animal species categories (*p* > 0.05). However, there were differences in tick infestation prevalence across different months and seasons, with the prevalence being highest in October (89.2%) and November (90.4%) and during the wet season (87.7%). Nonetheless, the difference in tick infestation prevalence between the seasons was not significant (*p* > 0.05).

Statistical analysis indicated that there was no significant difference in tick density between animals from Kebribayah and Afdem (*p* = 0.158) or between males and females (*p* = 0.603). However, age was significantly associated with tick density, with adult animals having a higher number of ticks than young animals (*p* = 0.007). There was no significant difference in tick density between animals with different BCSs (*p* = 0.877).

Animal species were similarly found to be significantly associated with increased tick density, with camels having a higher number of ticks than cattle (*p* = 0.014). Tick density varied depending on the recorded months and season, with the highest mean tick density occurring in November (32.69) and during the wet season (28.56). These factors were significantly associated with tick density (*p* < 0.001) (Table 8).

## 4. Discussion

The current study revealed a high prevalence of ticks among camels and cattle in the study area, with an overall prevalence of 85.1%. This is consistent with the previous studies conducted in Ethiopia [26, 27]. A total of 85.1% of camels and 85.1% of cattle in the study area were infested with ticks. In comparison, similar prevalence rates of 82.8% of tick infestation were reported from dromedaries in eastern Ethiopia by [28], and a prevalence rate of 81.3% was recorded for tick infestation in cattle in northwestern Ethiopia [29]. In contrast, dromedaries in the southern zone of Tigray have been reported to have a higher prevalence of hard tick infestations (96.6%) [30]. Similarly, [31] reported a prevalence of 98.2% for tick infestation in cattle in Southern Ethiopia. The variation in these results may be due to differences in agroecology, sampling season, acaricide application, and management practices among camel herders in the various study areas.

The tick species discovered in this study have been previously reported in camels and cattle in various parts of the country, including camels [30, 32–34] and cattle in Southwestern Ethiopia [35]. A total of 20,610 adult ticks were collected and identified as belonging to the three different genera: *Rhipicephalus*, *Amblyomma*, and *Hyalomma*. This aligns with the findings of [30, 33], who reported similar genera in the Tigray and Borane regions of Ethiopia. In the current study, *R. pulchellus* was the predominant tick species found on camels and cattle, comprising 48.9% of the ticks. This was followed by *A. gemma* at 26.3%, *H. truncatum* at 11.6%, *A. lepidum* at 6.7%, and *A. variegatum* at 6.5%. It is worth noting that previous studies have also reported a higher prevalence of *R. pulchellus* in camels, with prevalence rates of 70.47% [32], 85.2% [34], and 27.86% [30]. *R. pulchellus* has been reported as the most common tick species in cattle [31], with a prevalence of 75.2%. The wide prevalence of *R. pulchellus* can be attributed to its distribution in climatic regions such as savannas, steppes, and deserts. Moreover, it is the most frequently encountered tick species in Northeast Africa and Rift Valley regions [25].

The results of this study revealed that among all the species, female ticks were more abundant than males, except for *A. gemma* and *A. lepidium*. This finding is consistent with [26, 33], who conducted similar studies on different domestic animals. The higher number of female ticks found in animals can be attributed to their increased need for blood to produce eggs and their longer lifespan relative to male ticks, resulting in prolonged attachment periods. However, in certain species within the *Amblyomma* genus, female ticks may detach from the host after becoming fully engorged to lay eggs, while male ticks may continue feeding and mating for extended periods. In some cases, female ticks of these species may also attach to the host's skin in response to aggregation pheromones produced by feeding males [36].

This study identified a statistically significant correlation (*p* < 0.05) between the prevalence of tick infestation and the body condition score of camels and cattle, with animals in poor condition having a higher rate of infestation. These results are consistent with the findings of [37] but contradict those reported by [30]. In contrast, no significant variation in the prevalence of tick infestation was observed between the different areas of sampling (PAs). This observation is in line with the results reported by [32] for camels and [26] for cattle but appears to contradict the findings of [38] in the Jigjiga Zone, which suggested that tick infestation rates were influenced by the animals' living environment. Temperature and relative humidity are crucial ecological factors that influence the distribution and abundance of ticks in a given environment [39]. The absence of variation in the present study may be due to similarities in climatic conditions in the sampled peasant associations [40].

Additionally, the results of this study indicated that the prevalence of tick infestation was significantly higher (*p* < 0.05) in adult animals (90.8%) than in young animals (80.6%). This finding contradicts that of [29]. The higher prevalence of ticks in adult animals may be explained by their increased mobility and greater chance of encountering other animal species, resulting in greater exposure to ticks and an increased risk of tick infestation. However, there was no statistically significant difference (*p* > 0.05) in tick infestation rates between the male and female hosts. This finding is consistent with previous research conducted by [41] in Sudan and [42] in Iran on camels, as well as [25] in Belgium on cattle. Tick infestation varied significantly by month and season, with the highest prevalence in October (89.2%) and November (90.4%) and during the wet season (87.7%), although ticks were found on cattle throughout the study period. Tick infestation is known to persist all year round, although density may increase during wet seasons [43]. The study reported a significant increase in tick counts during the rainy season compared to the dry season [44].

The study found that tick density was influenced by age and animal species, as adult animals and camels had a greater number of ticks (*p* < 0.005). These findings are consistent with prior research conducted on camels [32, 33]. However, this contradicts the findings of [45], which reported a high number of ticks in young cattle. The study revealed that tick density varied depending on the recorded month and season, with November and the wet season having the highest mean tick density (*p* < 0.001). These findings are consistent with the results of previous research on camels by [34] and cattle by [45].

The present study indicated that tick infestation was the most common ectoparasitic disease, followed by mange and pediculosis. These findings align with those of previous studies conducted in the same area by [7, 46]. The results of the proportional pilling with informant groups indicated that young animals, both camels and cattle, had high tick burdens, followed by camels and cows. While farmers did not physically count the number of ticks on their animals, they accurately estimated the tick burden based on its impact on the growth and general well-being of the animals. This is particularly important in young and physiologically vulnerable animals, such as females, because the impact of ticks on their health can be severe [47].

According to the seasonal calendars used in this study, key informants in both districts agreed that tick infestation outbreaks typically occurred during the wet season. This finding is consistent with the results of conventional investigations of tick infestations, which have shown that the wet season is the most conducive period for tick outbreaks in livestock. The high humidity during this season creates a suitable environment for ticks to breed, feed, and lay eggs, leading to an increase in the tick population [48].

One limitation of the study is that it focused only on two districts within the Somali Regional State of Ethiopia, and therefore, the findings may not be generalizable to other areas. Additionally, the study did not investigate the economic impact of tick infestations on livestock production and the livelihoods of farmers. The study also relied on self-reported data from farmers, which may have introduced bias in the results. Finally, the study did not investigate potential environmental factors that may contribute to tick infestations in the area.

Based on the findings of this study and similar studies from the region, there is an urgent need to develop and implement tick control programs that are specific to the management and production environments of each district and that take into consideration the most effective tick control methods identified by livestock owners. We recommend the following: “Conduct further research to understand the economic impact of tick infestations on livestock production in the study area and to determine the best strategies for minimizing these losses. Provide education and training to livestock owners on the importance of tick control and the most effective methods to improve the adoption of recommended practices. Implement tick control measures twice annually during the wet seasons to target the highest tick populations, with a focus on reducing the burden of tick infestation on livestock.”

## 5. Conclusion and Recommendations

The current study found a high prevalence of tick infestation in camels and cattle in the study area, with an overall prevalence of 85.1%. This prevalence was slightly higher than that reported in previous studies in Ethiopia on tick infestation in dromedaries and cattle, but this may be attributed to location, sampling season, and management practices. *R. pulchellus* was identified as the most common tick species, followed by *A. gemma*, *H. truncatum*, *A. variegatum*, and *A. lepidum*. Female ticks outnumbered males, which could be attributed to the mating and feeding habits of these species. The prevalence of tick infestation varied significantly based on the condition score of the animals, with animals with poor BCS having a higher prevalence. However, there was no significant variation in tick infestation rates based on the sampling area or the sex of the host animal. The informant groups identified tick infestation as a priority disease in the area, with topical acaricide treatment options being the most effective control method. Additionally, outbreaks of tick infestation occur mostly during the wet season, when humidity and temperature are high. This study calls for increased awareness and management practices to control tick infestation in animals to ultimately improve household food security.

## Figures and Tables

**Table 1:** Name of FGDs assessed in kebeles of the two districts (Kebribayah and Afdem).

	Kebribayah District	Afdem District
Name of FGDs assessed in the kebeles	FGD1 (Guyow kebele)	FGD5 (Deladu kebele)
	FGD2 (Durwale kebele)	FGD6 (Darela kebele)
	FGD3 (Gumar kebele)	FGD7 (Ruqi kebele)
	FGD4 (Jingada kebele)	FGD8 (Quranjale kebele)

**Table 2:** Mean score and rank of three major ectoparasites determined by Kebribayah and Afdem livestock keepers through proportional piling exercises.

Ectoparasites	Kebribayah	Afdem	Rank
	Mean ± SD score	Mean ± SD score	
Ticks (Shilin)	57.0 ± 6.218	63.50 ± 5.508	1
Mites (Cadho)	28.50 ± 1.915	24.0 ± 5.657	2
Lice (Injir)	14.50 ± 5.260	15.0 ± 10.00	3
	*W* = 1 (*p* < 0.018)	*W* = 0.813 (*p* < 0.039)	

**Table 3:** Mean score on different age and sex categories for tick burden in camels and cattle determined by Kebribayah and Afdem livestock keepers through proportional piling exercises.

Animal category	Kebribayah	Afdem	Rank
	Mean ± SD score	Mean ± SD score	
Young camel	23.00 ± 6.27	23.00 ± 4.76	2
She camel	16.25 ± 4.79	17.25 ± 3.50	3
Adult camel	9.50 ± 4.20	8.50 ± 1.29	6
Young cattle	23.50 ± 4.73	25.75 ± 4.65	1
Cow	16.50 ± 4.73	14.00 ± 5.88	4
Adult cattle	11.25 ± 2.99	11.50 ± 1.92	5
	*W* = 0.788 (*p* = 0.008)	*W* = 0.640 (*p* = 0.025)	

**Table 4:** Mean score of tick control options based on their effectiveness in camel and cattle determined by Kebribayah and Afdem livestock keepers through proportional piling exercises.

Tick control option	Kebribayah		Afdem	
	Mean ± SD score	Rank	Mean ± SD score	Rank
Topical acaricides	42.50 ± 6.46	1	40.00 ± 4.082	1
Ivermectin	20.00 ± 9.13	3	23.75 ± 4.787	2
Manual removal	16.25 ± 7.50	4	18.75 ± 4.787	3
Integrated control of ticks	21.25 ± 8.54	2	17.50 ± 6.455	4
	*W* = 0.678, *p* = 0.043		*W* = 0.711, *p* = 0.036	

Topical acaricides include daizinon, amitraz, and DDT (dichloro-diphenyl-trichloroethane).

**Table 5:** Mean score on seasonal calendars for tick burden in camel determined by Kebribayah and Afdem livestock keepers through proportional piling exercises.

Season	Afdem		Kebribayah	
	Mean ± SD score	Rank	Mean ± SD score	Rank
Spring	45.00 ± 10.801	1	51.50 ± 8.888	1
Autumn	25.75 ± 9.946	2	21.75 ± 8.500	2
Summer	16.25 ± 16.008	3	15.50 ± 3.317	3
Winter	13.00 ± 7.257	4	11.25 ± 6.292	4
	*W* = 0.475, *p* = 0.127		*W* = 0.700, *p* = 0.038	

**Table 6:** Distribution and percent abundance of different tick species in camels and cattle in the study area.

Tick genera	Tick species	Male	Female	Total	Prevalence (%)
*Amblyomma*	*A. gemma*	101	5065	5166	26.3
	*A. lepidum*	1106	498	1604	6.7
	*A. variegatum*	1013	771	1784	6.5
*Hyalomma*	*H. truncatum*	1759	1675	3434	11.6
*Rhipicephalus*	*R. pulchellus*	4140	4482	8622	48.9
Total no. of ticks		8119	12,491	20,610	100

**Table 7:** Prevalence of tick infestation in camels and cattle in the study area in relation to various risk factors.

Variable	Category	No. examined	No. positive (%)	*χ* ^2^	*p* value
Animal origin	Kebribayah	450	375 (83.3)	2.24	0.13
	Afdem	450	391 (86.8)		

Sex	Male	254	220 (86.6)	0.63	0.43
	Female	646	546 (84.5)		

Age	Young	506	408 (80.6)	18.30	0.001
	Adult	394	358 (90.8)		

BCS	Good	217	156 (71.9)	40.615	0.001
	Medium	481	425 (88.4)		
	Poor	202	185 (91.6)		

Animal species	Camels	510	434 (85.1)		
	Cattle	390	332 (85.1)	0.00	0.99

Months	June	150	127 (84.7)	8.028	0.155
	July	154	133 (86.4)		
	Aug	151	120 (79.5)		
	Sep	245	206 (84.1)		
	Oct	65	58 (89.2)		
	Nov	135	122 (90.4)		

Season	Wet	350	307 (87.7)	3.063	0.080
	Dry	550	459 (83.5)		

**Table 8:** Analysis of associations between tick density and different host risk factors using a one-way ANOVA.

Variable	Category	No. positive (%)	Mean	SD±	95% CI		*F*	*p* value
					Lower bound	Upper bound		
Origin	Kebribayah	375 (83.3)	23.76	16.804	22.21	25.32	1.993	0.158
	Afdem	391 (86.8)	22.19	16.625	20.65	23.73		

Sex	Male	220 (86.6)	22.80	16.387	21.53	24.06	0.271	0.603
	Female	546 (84.5)	23.44	17.577	21.27	25.61		

Age	Young	408 (80.6)	21.65	16.887	20.17	23.12	7.379	0.007
	Adult	358 (90.8)	24.69	16.376	23.07	26.31		

BCS	Good	156 (71.9)	22.43	17.224	19.98	24.88	0.131	0.877
	Medium	425 (88.4)	23.15	17.102	21.65	24.65		
	Poor	185 (91.6)	23.07	15.358	20.97	25.16		

Animal species	Camels	434 (85.1)	24.54	18.049	22.74	26.33	6.007	0.014
	Cattle	332 (85.1)	21.79	15.549	20.43	23.14		

Months	June	127 (84.7%)	26.16	18.606	23.16	29.16	31.009	0.001
	July	133 (86.4%)	27.90	17.005	25.19	30.60		
	Aug	120 (79.5%)	17.00	11.759	15.11	18.89		
	Sep	206 (84.1%)	15.59	8.327	14.54	16.64		
	Oct	58 (89.2%)	25.54	16.492	21.45	29.63		
	Nov	122 (90.4%)	32.69	21.750	28.99	36.39		

Season	Wet	307 (87.7%)	28.56	19.750	26.49	30.64	68.682	0.001
	Dry	459 (83.5%)	19.42	13.323	18.31	20.54		

## Data Availability

Data are available upon request to the authors.
